# Equilibrium and Kinetic Studies of Phosphate Removal from Solution onto a Hydrothermally Modified Oyster Shell Material

**DOI:** 10.1371/journal.pone.0060243

**Published:** 2013-04-02

**Authors:** Jie Chen, Yun Cai, Malcolm Clark, Yan Yu

**Affiliations:** 1 College of Materials Science and Engineering, Fuzhou University, Fujian, China; 2 College of Materials Science and Engineering, Fuzhou University, Fujian, China; 3 School of Environment, Science and Engineering, Southern Cross University, Lismore, New South Wales, Australia; University of California, Berkeley, United States of America

## Abstract

Phosphate removal to a hydrothermally modified fumed silica and pulverized oyster shell material for use in wastewater treatments were made. Sorption data modeling (pH’s 3–11, P concentrations of 3, 5, 10, 15, 20, & 25 mg/L, and at an ambient temperature of 23°C) indicate that an optimal removal of P occurs at pH 11. Three kinetic models were also applied (a pseudo-first-order Lagergren kinetic model, a pseudo-second-order (PSO) kinetic and Elovich) and indicate that a PSO model best describes P-removal. In addition, an application of the Weber and Morris intra-particle diffusion model indicates that external mass transfer and intra-particle diffusion were both involved in the rate-determining step. Langmuir, Freundlich modeling of the sorption data also indicate that the heterogeneous Freundlich sorption site model best describes the data although Langmuir data also fit with data tailing suggesting data are not linear. The data collected indicates that the hydrothermally modified fumed silica and pulverized oyster shell material is suitable for use in wastewater treatment, with P-removal to the solids being preferential and spontaneous.

## Introduction

Contamination of potable ground water with phosphate and its removal in water treatment have become increasing focus worldwide. Many dephosphorization studies have been made for wastewaters, including biological, chemical precipitation, and adsorption processes [1]. Of all phosphate removal techniques, adsorption is receiving increasing attention and becoming an attractive technology because of its simplicity, low cost, ease of operation and handling, sludge free operation, and the capacity to regenerate and re-use solids. In this regard, many adsorbents have been explored such as: zeolite; modified-bentonite (Phoslock); bauxite refinery residues (red mud; Bauxsol™); calcined dolomite; fly ash; and ferric iron oxides [2]–[9].

With the rapid expansion of oyster cultivation in many coastal areas, an excess of oyster shell from shucking can not be used, which often leads to direct dumping at shoreline, roadside etc as a waste material that causes serious environmental pollution. However, full utilization of the calcium resource in oyster shell and the development of a highly efficient P removal material can not only reduce the environmental impact, but also change wastes into valuable resource. Moreover, oyster shells consist of three separate layers, a cuticle, prismatic, and nacreous layer in a particular configuration. The prismatic layer is dominant having a foliated texture that contains a great number of micropores [1], [10]–[12]. These natural pores can be utilized such that the oyster shell can have fairly strong absorbability, exchange capacity, and catalytic surface area that can be used for phosphorus removal from wastewaters.

We have previously reported on a calcined and hydrothermally annealed material shaped as a hollow cylindrical derived from oyster shell, which exhibited excellent phosphate removals [1]. We also characterized this material using XRD, SEM and EDS techniques to identify the crystalline phases and the microstructure evolution pre- and post- calcinations, hydrothermal annealing, and phosphate removal. It has been shown that CaSiO_3_ is produced during calcination that forms hydrated calcium silicates during hydrothermal annealing; these hydrated calcium silicates react with the soluble phosphate in wastewaters to precipitate a calcium phosphate. The SEM results also show an open microstructure was formed after calcinations and hydrothermal annealing process, which was benefit for adsorption [1]. In this study, the factors affecting phosphate adsorption and detailed information on equilibrium and kinetic removal properties of phosphate were investigated in order to optimize the adsorption process.

## Experimental

### 1. Materials

Oyster shells intended for waste disposal at Xiyangxincun market, Fuzhou City, were collected, cleaned, dried and ground for usage and the fumed silica was purchased from XIBEI Iron Alloy Company, China. For oyster shell powder (<200 mesh particle size) and fumed silica in the molar ratio of CaO/SiO_2_ was 5∶6, which provided an optimum weight ratio of 1∶1.39 g. After mixing as homogenous powder, cylindrical specimens (with a dry sample weight of 2.0 g) were formed [1], and were calcined at 800°C for 1 h before hydrothermal annealing at 150°C for 12 h.

### 2. Adsorption Experiments

Phosphate solutions at 3, 5, 10, 15, 20, & 25 mg/L were prepared from Na_2_HPO_4_^.^2H_2_O in de-ionized water. Solution pH was adjusted to 3, 5, 7, 9, or 11 using 0.1 M HCl or NaOH, and monitored using a pH electrode (Denver Instrument, Model 225, pHISE Meter) calibrated with standard buffer solutions.

Solution phosphate concentrations were determined using the ammonium phosphomolybdate blue method [13], with the absorbance was measured with an SP-721E spectrophotometer at 960 nm. Mass loading of the solids to solution was 1 g to 40 mL (25 g/L) and all the experiments were conducted at ambient temperature 23°C. To allow for any adsorption to the container surface, several control experiments without adsorbent were made, and showed that no adsorption occurred.

### 3. Equilibrium and kinetic Experiments

Equilibrium and kinetic experiments were performed at pH = 7, with initial concentrations fixed at 3, 5, 10, 15, 20, & 25 mg/L. The time to reach equilibrium was determined to be the time after which the solution concentration did not change significantly and was determined by kinetic adsorption study using sampling times of 3, 6, 9, 12, 36, 48, 72, 192 h, respectively. In order to generate an adsorption isotherm for P, the adsorption capacity of the oyster shell adsorbent (mg of P per g of adsorbent) was determined by calculating the mass of P adsorbed (mg) and dividing it by the weight of the adsorbent (g) for each different initial concentration (mg/L). The equilibrium sorption capacity (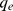, mg·L^−1^) and the removal ratio (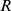, %) for P were determined using Equations (1&2):
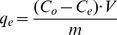
(1)

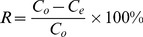
(2)where 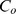 is the initial P concentration and 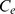 is the concentrations of P at the equilibrium time (mg/L); 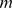
is the mass of adsorbent (g/L); and 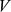 is the volume of the phosphate solution (L).

## Results and Discussion

### 1. Effects of the Initial Concentrations and pH Values on the P Adsorption

**Figure 1:**
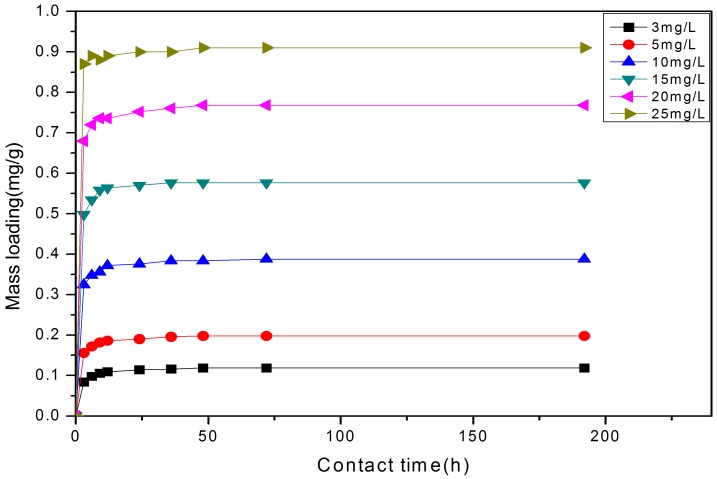
Effect of initial concentrations on the removal of P from solution to the solids. Data show that increasing concentration leads to an increased mass loading to the solids, but also as slight delay in reaching equilibration (e.g., 12 h at 3 mg/L, but nearly 48 h at 25 mg/L).

**Figure 2:**
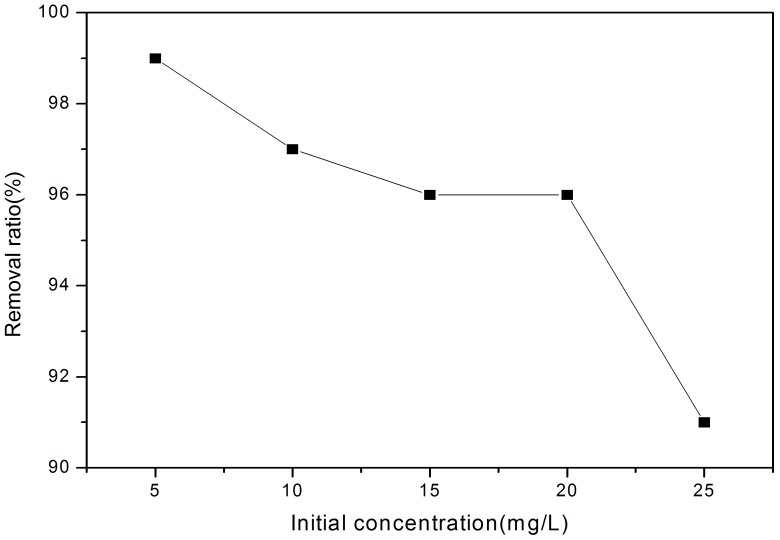
Removal ratio from solution to solids for increasing initial P-concentration. Data show that as solution concentration increases efficiency of the removal decreases, but efficiency remains>90% at 25 mg/L.

Figures (1 & 2) show the mass loading (mg/g) with time and removal ratio from solution for the experiments. As expected, the mass loading of P initially increased rapidly and then continued increasing at a slower pace until equilibrium was achieved, moreover these mass loadings show that the higher the concentration of P the greater the mass loading on the solids (Fig. 1). For the initial concentration of 3, 5, 10, 15, 20, & 25 mg/L, the equilibrium adsorption capacities of P ion were 0.119, 0.198, 0.388, 0.576, 0.768, 0.910 mg/g, respectively. However, although adsorbed P ion increased from 0.119 mg/L to 0.910 mg/L, the removal ratio decreased from 99.17% to 91.00% (Fig. 2), which may be due to the ratio between P ion and the available binding sites [14], [15]. When the initial P ion concentration was low, available binding sites were relatively higher, as described previously [14]–[18]. The removal ratio exceeded 90% for all concentrations, indicating that the material can provide significant P-removal for wastewaters across a wide concentration range. Furthermore, the removal equilibrium at each concentration was attained within 48 hrs, and the slope of the plots (Fig. 1) represents the initial removal rate. Figure 1 also shows that when the removal times lengthened that surface loading continued to increase, although at a much slower rate than the more rapid initial adsorption rate. This continual increase may be attributed to the abundant pores existing in the solids [1], which allows water and P infiltration within the pellet, thereby contributing to the adsorption capacity. Furthermore, wastewater with higher initial P-concentrations provided more P to meet the highly dynamic removal conditions in the initial removal stage; the very sharp P removals in these initial stages would suggest precipitation may be a key removal mechanism.

**Figure 3:**
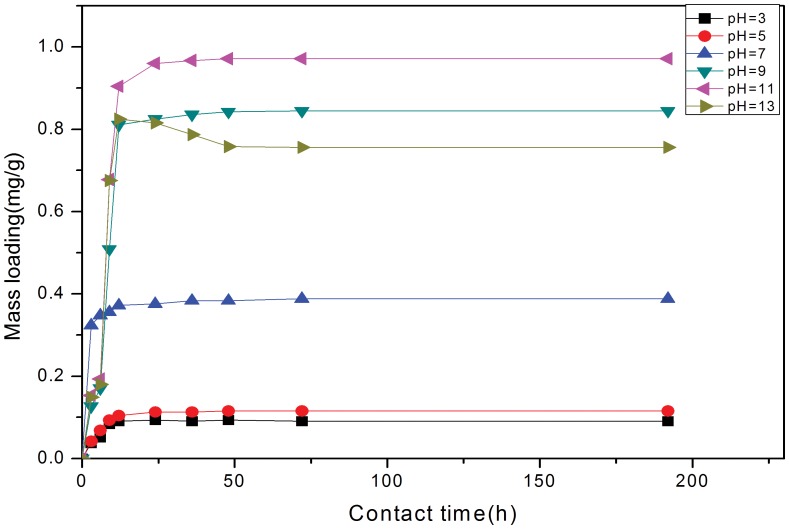
Effect of pH values on the mass loading of P to the solids for the 10 mg/L solution. For pH 9, 11 and 13, there appears to be 2 stages to the loading from 0–8 hours where surface sorption is the initial control with a precipitation from about 8 hours onwards. The pH 13 data show a re-solubilisation of precipitated P from 16–48 hours as equilibrium establishes.

The P removal results for 10 mg/L at different pH are provided in Figure 3, and show that pH had substantial effect on the P removal capacity. Under acidic conditions, removal capacity is substantially lower than for alkaline conditions and reaches a maximum value at pH 11. Consequently, with continuous raising the pH to 13, the mass loading could reach 0.971 mg/g at pH 11, which is a pH where HPO_4_^2−^ is the dominant species. This result between solution pH and mass loading correlates well with calcium phosphate precipitation [1]. In our preview work [1], we reported that during calcination at 800°C, CaCO_3_ was converted to CaO which partially fused with SiO_2_ to form CaSiO_3_, contributing to the active calcium ion distributing in crystal lattice particles that could react with free phosphate radicals in wastewater. Under neutral or alkaline condition, direct precipitation Ca_3_(PO_4_)_2_ and Ca_5_(PO_4_)_3_OH are readily achieved (Equations 3 & 4). Moreover, Ca_5_(PO_4_)_3_OH is the most thermodynamically stable and most difficult to solubilise [19]. However, under more acidic conditions, CaHPO_4_, Ca_4_H(PO_4_)_3_, and Ca_3_(PO_4_) are thermodynamically more stable. The precipitation of Ca_3_(PO_4_)_2_ and Ca_5_(PO_4_)_3_OH most likely follow as:
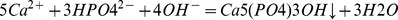
(3)

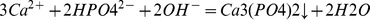
(4)Where increases in OH^−^ allow the chemical precipitations to occur more readily resulting to the higher removals. However, a large excess of OH^−^ would appear to impair Ca_5_(PO_4_)_3_OH precipitation allowing a dissolution back in to solution with increased reaction times (Fig. 3). Hence, the calcined and hydrothermally annealed oyster shell material is most effective in P immobilisation in the pH range of 9–11.

### 2. Adsorption Isotherm

Two most common models used to investigate, and describe solution removals processes and mechanisms are Langmuir and Freundlich models. The Langmuir isotherm model assumes a completely homogeneous surface, where the sorption onto the surface has the same activation energy [20],whereas the Freundlich isotherm model is suitable for highly heterogeneous surfaces [11].

#### 1) Langmuir isotherm

The Langmuir isotherm equation in its liner form can be expressed as follow:
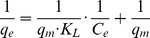
(5)where 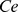 is the equilibrium concentration (mg/L), 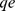 is the amount removed to the solid (mg/g), 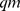 is the maximum saturation capacity at the isotherm temperature (mg/g), and 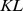 (L/mg) is the sorption equilibrium constant related to the energy of adsorption. 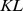 and 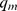 can be determined from the slope and the intercept in a plot of 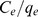 against 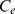. A dimension less constant separation factor (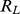) is defined based on the Equation 6 [21], [22]:
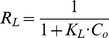
(6)where 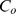 is the initial concentration of adsorbate (mg/L); 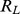 is considered as a more reliable indicator of the adsorption. There are four possibilities for the 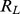 value: (i) for favorable adsorption, 0< 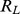
<1; (ii) for unfavorable adsorption, 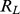
>1; (iii) for linear adsorption, 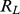 = 1; (iv) for irreversible adsorption, 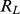 = 0 [22].

**Figure 4:**
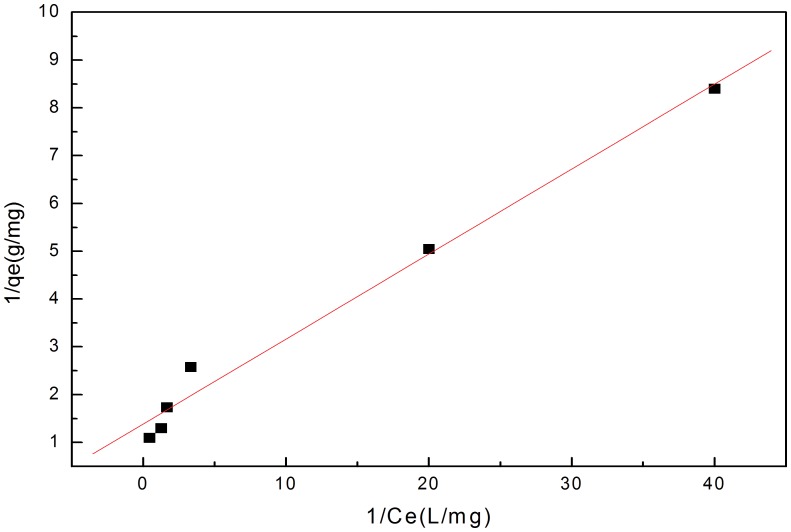
Linear regression of Langmuir equation at pH 7, a typical pH of waste waters.

**Figure 5:**
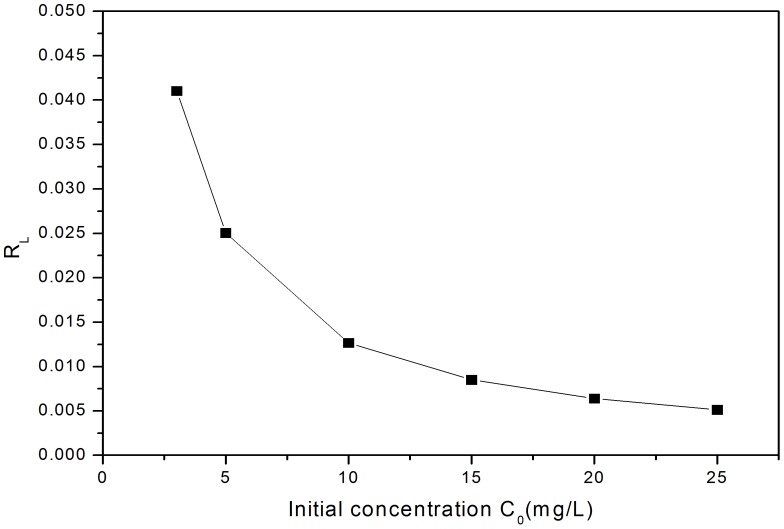
Plot of separation factor versus initial P ion concentration for the Langmuir model (Fig. 4) at pH 7.

The Linear regression of Langmuir equation (Fig.4) and the Langmuir constants calculated are shown in Table 1, and suggest the maximum saturation capacity that could reach at pH 7 is 0.722 mg/g (typical of waste waters), which is lower than the 0.971 mg/g seen at the sorption maxima at pH 11. A correlation coefficient for 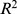
of 0.993 indicates that the removal process fits well with the Langmuir model, namely the adsorption behavior belonged to a single-layer adsorption. What’s more, the value of 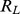 ranged from 0.005 to 0.041, indicating the adsorption was a favorable process (Fig. 5). However, despite the strong linear correlation, the Langmuir data indicate that the curve is 2 straight-line segments, with an intersection at about 5 (1/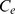 L/mg), which would suggest that surface sites are not homogeneous, or that 2 competing processes e.g., sorption and precipitation are occurring.

**Table 1:** The parameters, regression coefficients (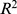) of the Langmuir and Freundlich model.

Langmuir			Freundlich		
*q_m_* (mg/g)	*K_L_* (L/mg)	*R* ^2^	*n*	*K_F_* (L/mg)	*R* ^2^
0.722	7.791	0.993	2.177	0.714	0.989

#### 2) Freundlich isotherm

**Figure 6:**
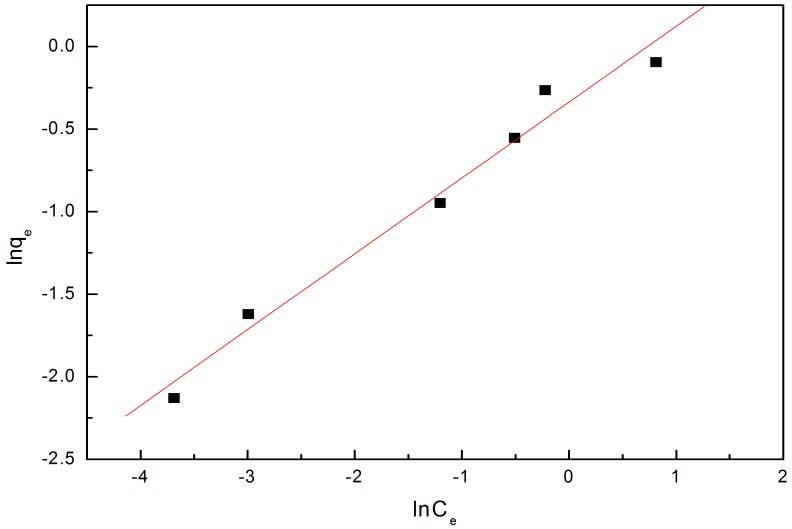
Linear regression of Freundlich equation at pH 7.

The Freundlich isotherm equation in its linear form can be expressed as:
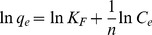
(7)where 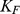 is the intercept, and 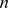 the derivative of the slope, are the Freundlich constants representing the adsorption capacity and the adsorption intensity respectively. In generally, the greater 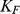, the greater the heterogeneity, and the larger the value of 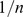 (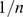
>1) more spontaneous the adsorption process became. The linear fitting curve (Fig. 6) and the calculated parameter (Table 1) gave an 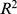 of 0.989, indicating that the Freundlich model was also in good agreement with the experimental data. Considering that there are two competing processes (phosphate adsorption & phosphate precipitation) to P-removal, the Freundlich offers the better description despite the slightly lower 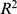, because the Freundlich model describes a heterogeneous site distribution, whereas the Langmuir model describes a homogeneous site model. The value of 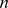 (2.177) was >1, indicates that P-removal to the solids was a preferential and spontaneous. Consequently, the adsorption of P at ambient temperature 23°C and pH 7 onto the oyster shell material could be described by both Langmuir and Freundlich model, but is probably better described by the heterogeneous Freundlich model.

### 3. Kinetic Models

In general, kinetic models are classified into two groups: a reaction model and the diffusion model [23].

#### 1) Reaction models

Three models were used to describe the removal of P from solution: a pseudo-first-order or Lagergren kinetic model; a pseudo-second-order (PSO) model; and Elvoich model. The linear forms of these three models can be represented by Equations (8, 9 & 10), respectively:
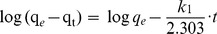
(8)

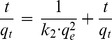
(9)

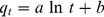
(10)where 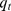 (mg/g) and 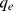 (mg/g) are the solid loadings at time t and at equilibrium, respectively. 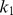
(min^−1^) is the pseudo-first-order rate constants for removal; 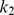
(g·mg^−1^·min^−1^) is the PSO rate constant; 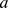 and 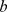 are Elovich constants.

**Figure 7:**
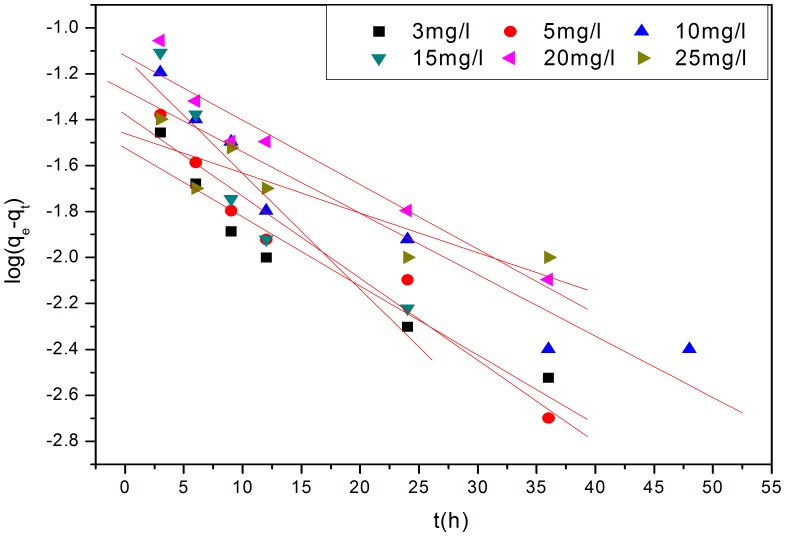
Plots of the pseudo-first-order Lagergren model for the adsorption of P ion. The poor data to fits with linear regression lines (Table 2) suggests that a pseudo-first-order Lagergren model is unlikely to control P-removal.

**Figure 8:**
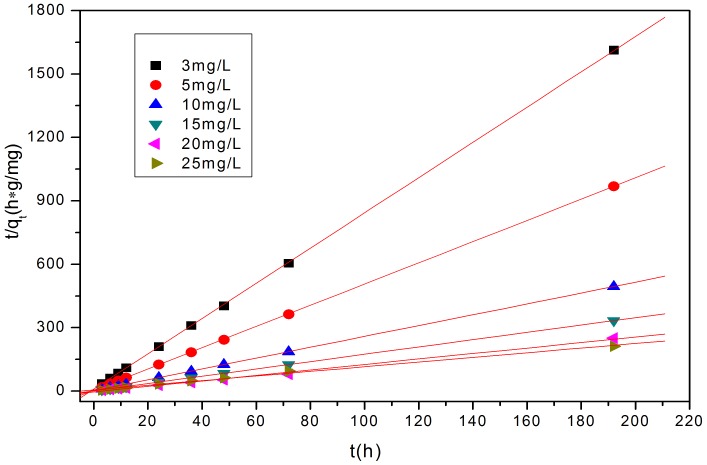
Plots of the pseudo-second order model for the removal of P to the modified oyster material. Strong correlations for data (Table 2) and agreement between experimental and calculated *Qe* data suggest that this model represents P-removal extremely well.

**Figure 9:**
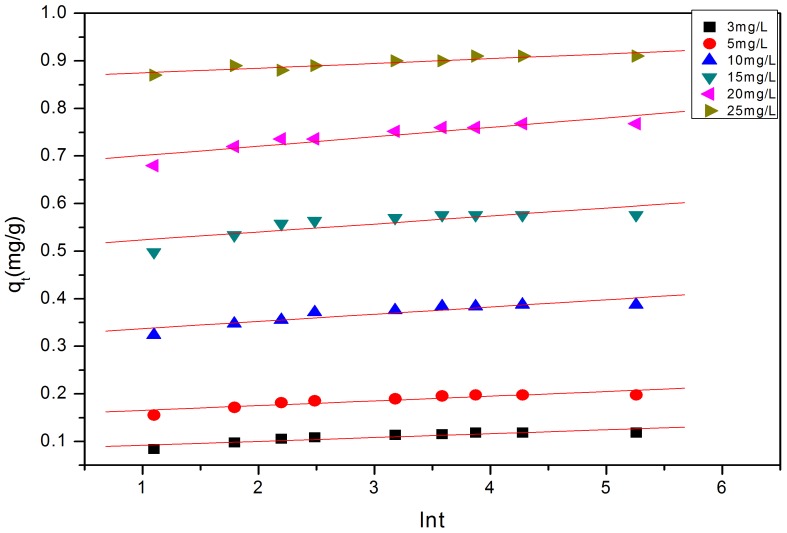
Elovich model plots for the removal of P to the modified oyster material. Data fit well to linear plots suggesting that, this kinetic model may describe P-removal.

Figures 7, 8 and 9 show the linear fitting curves for the three kinetic models, respectively, where data to generate the kinetic reaction plots was obtained from Figure 1 (see Section3.1.1.); Table 2 lists the parameters and regression coefficients (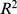). It can be seen from three plots, and from data in Table 2, that the PSO model provided better correlation coefficients than the other two models with the regression coefficients 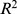
>0.999 for all initial concentrations investigated. Meanwhile, the equilibrium removal capacity calculated depending on the PSO rate model (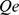, cal) was much closer to the experimental data (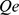, exp; Table 2) indicating that the PSO rate model provides very good summary of P removal from solution to the oyster shell material. This is significant because the PSO rate model assumes a chemically rate-controlling removal process [24]. However, the PSO includes the whole removal procedure, such as, precipitation, co-precipitation, external film diffusion, surface adsorption and intra-particle diffusion, being compatible with the analysis results below [25]; data presented here is in good agreement with the theory above.

**Table 2:** The parameters, regression coefficients (*R* ^2^) of the adsorption kinetic models; *Qe* (exp) means the adsorption capacity from the experiments while *Qe* (cal) from the calculation by equations.

C_0_	Pseudo-first-order kinetic model				PSO kinetic model				Elovich model		
	*K*_1_	*R* ^2^	Qe(exp)	Qe(cal)	*K*_2_	*R* ^2^	Qe(ca)	*h*	*R* ^2^	a	b
3	0.05	0.96	0.119	0.218	5.88	0.999	0.121	0.086	0.89	0.01	0.08
5	0.06	0.97	0.198	0.252	5.07	0.999	0.201	0.205	0.90	0.01	0.15
10	0.05	0.95	0.388	0.280	3.32	0.999	0.391	0.508	0.90	0.02	0.31
15	0.05	0.93	0.576	0.323	4.21	0.999	0.580	1.415	0.76	0.02	0.49
20	0.04	0.97	0.768	0.326	2.53	0.999	0.771	1.505	0.88	0.03	0.67
25	0.03	0.89	0.91	0.232	4.13	0.999	0.913	3.443	0.87	0.01	0.86

For the PSO model, the initial sorption rate could be obtained from Equation (11) [20]:
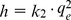
(11)


The PSO constants 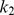 (Table 2) obtained from plotting (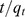) against 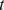, and the value of 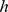 increased according to the increased initial concentration. The data indicate that similar observations made from plot slopes (Fig. 1; Section 3.1.) are present for the kinetic models that there appears to be a 2 stage process with an increasing rate of removal with increased concentration in the initial stage of removal.

#### 2) Diffusion models

The sorption process can be described by four-consecutive steps [18], [20], [23], [24], [26]–[29]:

  1. transport in the bulk of the solution;
  2. diffusion through the solution to the external surface of the adsorbent (also called film mass transferor boundary layer diffusion of solute molecules);
  3. particle diffusion in the liquid contained in the pores and in the sorbate along the pore walls;
  4. sorption and desorption within the particle and on the external surface.

Generally, steps 1 and 4 occur rapidly so that the rate-controlling steps becomes step 2, step 3, or the combination of them. The PSO model cannot identify the diffusion mechanism, hence to determine the rate-controlling step for P-removal to the oyster shell material, the Weber and Morris intra-particle diffusion model was introduced. The Weber and Morris intra-particle diffusion model was derived from Fick’s second law of diffusion and is expressed as [15], [28], [29]:
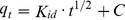
(12)where 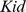 (mg.g^−1^.min^−1/2^ ) is the intra-particle diffusion constant, and is derived from the slope of the plot 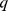 vs. 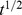. 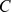 (mg/g) is the intercept of the plot, often referred to the thickness of the boundary layer [30], and a large 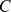 value is indicative of external mass transfer as being significant in the sorption, thereby acting as the rate-controlling step. In addition, when data fitting is linear, intra-particle diffusion is involved in the sorption process and when the fit passes through the origin (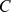 = 0), intra-particle diffusion is rate-limiting step [24], [28].

**Figure 10:**
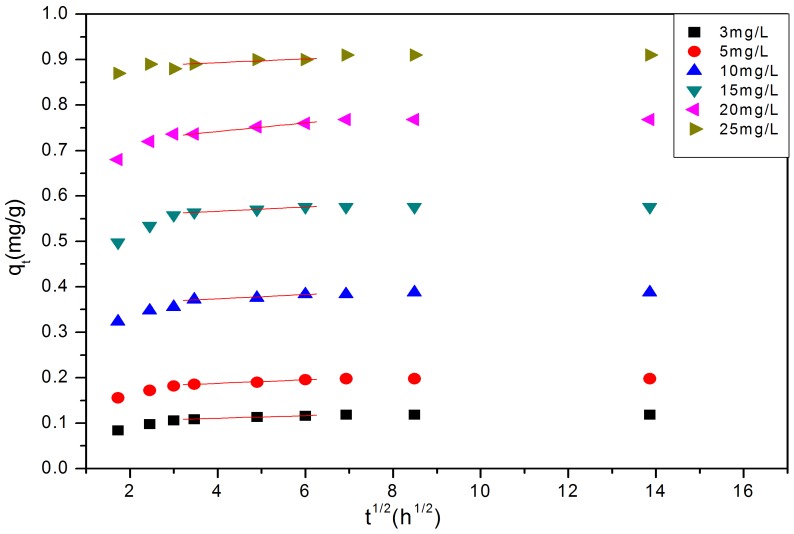
The linear regression of part data points.

Curve fitting of Weber and Morris intra-particle diffusion model (Fig. 10) shows that all the plots were similar, and that curves have two parts. The first steeper curved portion to the break in slope at 3–3.5 (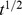
(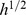)), is regarded as being a rapid external mass transfer, the second oblique linear, is regarded as the intra-particle diffusion portion; a third section (not clearly evident in our data) is a final plateau portion, where the removal process tends to equilibrium. Linear fitting of the oblique linear section (Fig.10) provides reliable 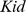 and 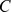 estimates (Table 3), high correlation coefficient 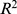. These data (Fig. 10, Table 3) indicate that the intra-particle diffusion was involved in rate limiting P-removal, however no fits pass through the origin, and therefore intra-particle diffusion was not the sole rate-controlling step during the gradual adsorption (Fig. 1); rather a combination with external mass transfer. The intra-particle diffusion rate constant 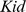 shows an increase in the adsorption rate increasing with increasing of initial concentrations. These data are consistent with diffusion theory [15], [28], [29], and is internally consistent with data collected. Diffusion is driven by the concentration gradient that develops between the surface and intra-particle sorption sites within the crystal lattice. Higher surface loading (Fig. 1) show that surface loadings are highest at higher solution loadings, and hence a greater concentration gradient develops to drive diffusion from the surface to deeper crystal lattice position and typically leads to a greater irreversibility of binding (see Clark et al [31], [32]). Moreover, a decrease in the efficiency in the P-removal (Fig. 2) shows that there is an increased residual solution concentration, which also sets up a second diffusion gradient between solution and surface, which further encourages the diffusion to occur. However at the highest P-concentration, there is a significant decrease in P-removal at 8 h, indicating a dissolution/desorption occurs. This is particular evident at high pH 13 and this dissolution/desorption lowers 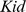 at higher concentrations (Table 3).

**Table 3:** The intra-particle diffusion rate constants and intercept values.

	3 mg/L	5 mg/L	10 mg/L	15 mg/L	20 mg/L	25 mg/L
*R* ^2^	0.986	0.982	0.965	0.997	0.993	0.901
*C*	0.09961	0.17206	0.35514	0.54747	0.70362	0.87707
*K*_id_ (mg.g^−1^.min^−1/2^)	0.00280	0.0039	0.0046	0.0047	0.0096	0.00409

### Conclusion

Experimental results of this study indicate that the oyster shell material is an effective adsorbent for phosphate removal from wastewater. Equilibrium is obtained rapidly within 48 hrs with removal ratio exceeding 90% for all initial concentrations. P removal is highly pH- and concentration dependent, where alkaline condition (pH = 9–11) are more conducive to removal; P removal maximum is at pH = 11.

Langmuir and Freundlich isotherm models both give high *R* ^2^ values, however, competing removal processes suggest that the heterogeneous surface distributions of the Freundlich model are the better fit. Moreover, the kinetics of P-removal is best described by pseudo-second-order rate model, where the implied rate-controlling process is the Weber and Morris intra-particle diffusion model. Consequently, the P-adsorption process is complex mixture of intra-particle diffusion and external mass transfers that have a combined impact on the rate-controlling process.
